# The GNL3L‐MDM2 Interaction Drives Esophageal Squamous Cell Carcinoma Progression

**DOI:** 10.1002/cam4.71146

**Published:** 2025-08-26

**Authors:** Aijie Yang, Haiyun Song, Yufeng Cheng

**Affiliations:** ^1^ Department of Radiotherapy Qilu Hospital of Shandong University (Qingdao) Qingdao China; ^2^ Department of Pathology Qilu Hospital of Shandong University (Qingdao) Qingdao China; ^3^ Department of Radiation Oncology Qilu Hospital of Shandong University Jinan China

**Keywords:** apoptosis, esophageal squamous cell carcinoma, GNL3L, invasion, MDM2, proliferation

## Abstract

**Background:**

This study investigates the mechanisms by which *GNL3L* influences ESCC progression.

**Methods:**

*GNL3L* expression was analyzed via immunohistochemistry in ESCC tissues. Cell proliferation (EdU and CCK8 assays), migration, invasion (wound healing and Transwell assays), cell cycle, and apoptosis (flow cytometry) were assessed. Levels of GNL3L, MDM2, p53, and p21 were evaluated by qRT‐PCR and western blot. Tumor growth was observed in nude mice injected with TE‐1 cells.

**Results:**

*GNL3L* was upregulated in ESCC specimens (*p* < 0.05) and knockdown reduced proliferation and migration while enhancing apoptosis (*p* < 0.01). GNL3L interacted with MDM2; knocking down *GNL3L* decreased MDM2 and increased p53 and p21 (*p* < 0.01). *MDM2* overexpression enhanced malignant characteristics, reversible by *GNL3L* silencing (*p* < 0.01). Moreover, *MDM2* knockdown inhibited malignant characteristics, reversible by *GNL3L* overexpression (*p* < 0.01). In vivo, the sh‐GNL3L group exhibited the smallest tumor volumes after 5 weeks (*p* < 0.01).

**Conclusions:**

*GNL3L* correlates with ESCC malignancy, influencing the MDM2‐p53‐p21 axis. GNL3L‐MDM2 interaction is critical in ESCC progression.

## 1 Introduction

Esophageal cancer (EC), one of the most prevalent malignant tumors, is notorious for its high morbidity and mortality, ranking as the seventh leading cause of cancer‐related deaths worldwide, with the highest incidences being observed in Eastern Asia and Eastern Africa [1]. The most common histological subtype is esophageal squamous cell carcinoma (ESCC), which is predominant in Asia and accounts for 90% of all EC cases in China [2]. Although non‐invasive screening and endoscopic techniques enable early detection and treatment measures, nearly half of ESCC patients are still diagnosed with unresectable or metastatic tumors [3]. In addition, ESCC has poor sensitivity to chemotherapy, and even targeted drugs fail to significantly improve survival rates [4]. Hence, the 5‐year overall survival of patients with ESCC is 18.9% [1, 5]. Therefore, the molecular mechanisms of ESCC must be clarified to provide novel treatment targets and new biomarkers for ESCC prognostication.

Guanine nucleotide‐binding protein‐like 3‐like (*GNL3L*) is an evolutionarily conserved GTP‐binding nucleoprotein that belongs to the HSR1‐MMR1 subfamily of GTPase [6]. In recent years, the roles of *GNL3L* in cell proliferation, invasion, migration, and apoptosis have received increasing attention [7, 8, 9]. In addition, the effects of *GNL3L* on tumorigenesis and progression of human glioblastoma multiforme and colorectal cancer have been demonstrated [10]. *GNL3L* can bind E3 ubiquitin ligase mouse double minute 2 (*MDM2*) in vivo and stabilize the *MDM2* protein to prevent its ubiquitination [11]. *MDM2* is the most critical *p53* negative regulatory factor and can bind and catalyze the polyubiquitination of p53, thereby promoting its degradation through the proteasome pathway [12]. *p53* is an important tumor suppressor, and its response genes include those that induce apoptosis and cell cycle inhibition, such as *p21* [13, 14]. The enhanced degradation of p53 promotes cancer progression [15]. Dai et al. [8] showed that *GNL3L* expression was upregulated in ESCC, but they did not examine *MDM2* or *p53*; they also examined *GNL3L* expression in human specimens and did not explore the mechanisms in vitro.

Hence, the purpose of the present study was to investigate the effect of *GNL3L* on ESCC cell proliferation, invasion, migration, and apoptosis; elucidate the underlying mechanisms; and provide new strategies and a corresponding theoretical basis for targeted therapy and prognostication in ESCC.

## 2 Materials and Methods

### 2.1 Bioinformatics

*GNL3L* expression was analyzed in EC tumor tissues and normal esophageal tissues using the Gene Expression Profiling Interactive Analysis (GEPIA) database [16], which includes 182 EC and 286 normal tissue samples from The Cancer Genome Atlas (TCGA) and Genotype‐Tissue Expression (GTEx). The threshold set for significant differences was log2|fold change| ≥ 1 and *p* < 0.01. A Kaplan–Meier analysis of survival probability was performed in EC patients with low expression of *GNL3L* (*n* = 138) and high expression of *GNL3L* (*n* = 46). The data were downloaded from the University of Alabama at Birmingham Cancer Data Analysis Portal (UALCAN) website [17]. The patient cohort was also obtained from the gene information of TCGA EC datasets.

### 2.2 Clinical Specimens

Surgical paraffin‐embedded specimens from 54 patients with ESCC who underwent surgery between January 2018 and December 2020 were obtained from the authors' hospital. The inclusion criteria were (1) European Cooperative Oncology Group (ECOG) performance status (PS) 0–2 [18], (2) no cancer treatments before surgery, (3) age < 80 years, (4) no distant metastases, and (5) complete surgical resection (R0 resection). Patients with other primary cancers were excluded. Four patients were female, and 50 were male. They were 48 to 77 years old. The expression of GNL3L in ESCC was analyzed by immunohistochemistry. This study was conducted in compliance with the Declaration of Helsinki and approved by the Ethics Committee of Qilu Hospital, Shandong University (Qingdao). Written informed consent was obtained from all patients.

### 2.3 Immunohistochemistry

Immunohistochemistry was performed as previously described [19]. Tissue sections were incubated overnight at 4°C with GNL3L antibody (1:500, Affinity, DF4113, Jiangsu, China), followed by incubation for 30 min at 37°C with a secondary antibody goat anti‐rabbit IgG (H + L) (1:5000, Affinity, S0001, Jiangsu, China). The sections were incubated with the avidin‐biotin‐peroxidase complex for 60 min at 37°C, and diaminobenzidine (DAB, Beijing, China) was used to develop peroxidase activity. Hematoxylin was used to counterstain the sections. PBS was used as a substitute for the primary antibody as a negative control. Five fields were observed from each section with high power, and the positive signals were counted. Following the two‐way scoring system, the final scores were calculated by multiplying the staining intensity by the percentage of stained cells. The percentage of stained cells was 0 (0%), 1 (1%–25%), 2 (26%–50%), 3 (51%–75%), and 4 (76%–100%). The staining intensity was evaluated as follows [20]: 0 (no staining), 1 (weak staining), 2 (moderate staining), and 3 (strong staining). There were four types of evaluations for the samples: no expression (final score of 0), low expression (final score ≤ 3), moderate expression (final score of 4–8), and high expression (final score ≥ 9).

### 2.4 Cell Lines and Reagents

ESCC (TE‐1, KYSE‐410, KYSE‐30, EC9706, and ECA‐109) and human normal esophageal epithelial (HEEC) cell lines were obtained from Procell Life Science &Technology Co. Ltd. (Wuhan, China). The cells were cultured at 37°C with 5% CO_2_ in Roswell Park Memorial Institute 1640 medium (RPMI‐1640, Procell, PM150110, Wuhan, China) with 10% fetal bovine serum (FBS, Procell, 164,210, Wuhan, China). Cells were passaged when they grew to 70%–80% confluence.

### 2.5 Cell Transfection

When reaching 40%–60% confluence, the cells were inoculated in six‐well plates and incubated for 24 h. Cells were treated with shRNA‐GNL3L (sh‐GNL3L), shRNA‐MDM2 (sh‐MDM2), pcDNA‐MDM2 (oe‐MDM2), or pcDNA‐GNL3L (oe‐GNL3L) in the presence of Lipofectamine 2000 (Invitrogen) and incubated for 48 h. The transfection efficacy was measured using quantitative real‐time polymerase chain reaction (qRT‐PCR) and western blotting.

### 2.6 Cell Counting Kit‐8 (CCK‐8) Assay

The CCK‐8 assay (Procell, P‐CA‐001, Wuhan, China) was used to evaluate the proliferation of the cells. After transfection, 2 × 10^3^ cells per well were cultured in a 96‐well plate. At 24, 48, and 72 h, the supernatants were removed, and 100 μL of CCK8 solution (10 μL CCK8 and 90 μL RPMI‐1640 medium) was added to each well and incubated for 3 h. Absorbance was measured using a microplate reader at 450 nm. Each experiment was performed in triplicates.

### 2.7 5‐Ethynyl‐2‐Deoxyuridine (EdU) Assay

After cell transfection, 100 μL of EdU medium was added to each well, and the cells were washed with PBS. Cell fixation, cell permeability, and DAPI staining were carried out according to the manufacturer's instructions using the EdU Cell Proliferation Imaging Assay Kit (Procell, P‐CA‐047, Wuhan, China). The percentage of EdU^+^ cells was calculated using the equation: EdU‐positive rate = (EdU^+^ cells)/ (DAPI‐stained cells) × 100%.

### 2.8 Wound Healing Assay

The wound healing assay was conducted in six‐well plates. After manually scratching the monolayer of cells, RPMI 1640 with 10% FBS was replaced, and the cells were washed in a medium without FBS. Wound closure was recorded to determine the proportion of wound healing after 24 h (magnification 40×).

### 2.9 Transwell Assay

After transfection, the Transwell migration and invasion assays were conducted using Transwell chambers (Corning, USA). For the Transwell invasion assay, the bottom of the Transwell chambers was coated with Matrigel for 24 h, and 200 μL (2.0 × 10^5^/ml) of cell suspension was added to the upper chamber before the cell suspension was inserted into the 24‐well chamber. Afterward, the cells at the bottom were fixed in 4% paraformaldehyde for 15 min, stained with crystal violet for 20 min, and counted under a microscope. For the Transwell migration assay, Matrigel was not coated on the Transwell membrane. Finally, the migrated and/or invasive cells were counted utilizing ImageJ software (National Institutes of Health, Bethesda, MD, USA).

### 2.10 Cell Apoptosis and Cell Cycle Analysis

After transfection, the cells were prepared for 48 h in six‐well plates with 2.0 × 10^5^ cells per well. PBS was used for collection, and the cells were washed twice with PBS at 4°C. The Cell Cycle and Apoptosis Analysis Kit (Beyotime, C1052, Shanghai, China) and Annexin V‐FITC Cell Apoptosis Analysis Kit (Beyotime, C1062M, Shanghai, China) were used for cell cycle and apoptosis analyses, respectively. Cell apoptosis and cell cycle distribution were analyzed on a FACScan cytometer using the FlowJo Software.

### 2.11 Quantitative Real‐Time PCR (qRT‐PCR)

For qRT‐PCR assays, Trizol Reagent (Invitrogen, 15596018CN, Carlsbad, CA, USA) was used to extract RNA. The Primescript RT Reagent (Takara, RR037A, Otsu, Japan) was used to reverse‐transcribe the RNA. The cDNA was subjected to qRT‐PCR with SYBRPremix Ex Taq (Takara, RR036A, Otsu, Japan). The relative level of each sample was determined using the 2^‐ΔΔCT^ method and normalized to glyceraldehyde‐3‐phosphate dehydrogenase (GAPDH) in triplicate. The primers were *GNL3L 5′‐AGC* AGC CTG ATC AAT AGC CT‐3′ (forward) and 5′‐TCC AAG AGC CGG ATG AAC TT‐3′ (reverse), *GAPDH 5′‐GAA* GGT GAA GGT CGG AGT C‐3′ (forward) and 5′‐GAA GAT GGT GAT GGG ATT TC‐3′ (reverse), and *MDM2 5′‐GAT* CCA GGC AAA TGT GCA ATA C‐3′ (forward) and 5′‐TGG TCT AAC CAG GGT CTC TT‐3′ (reverse).

### 2.12 Western Blot

Cells were lysed on ice, and total proteins were extracted using radioimmunoprecipitation assay (RIPA, Beyotime, P0013C, Shanghai, China) buffer containing protease inhibitor I (Beyotime, P1006, Shanghai, China). Protein concentrations were determined using the BCA protein assay kit (Beyotime, P0010, Shanghai, China). The proteins were separated using 10% sodium dodecyl sulfate‐polyacrylamide gel electrophoresis (SDS‐PAGE). The proteins were loaded onto PVDF membranes (Solarbio, Beijing, China) and blocked in 5% skim milk for 2 h. The membranes were incubated with the primary antibodies overnight at 4°C, followed by the secondary antibody for 1 h at room temperature. The primary antibodies were GAPDH polyclonal antibody (1:3000, Affinity, AF7021, 36 kDa), MDM2 polyclonal antibody (1:1000, Affinity, AF0208, 90 kDa), GNL3L antibody (1:1000, Affinity, DF4113, 66 kDa), p53 antibody (1:1000, Affinity, AF0879, 53 kDa), and p21 antibody (1:1000, MedChemExpress, HY‐P80774, 18 kDa). The secondary antibody was goat anti‐rabbit IgG (H + L) (1:5000, Affinity, S0001, Jiangsu, China). The Western Blotting ECL Substrate (Affinity, KF8005, Jiangsu, China) was used to visualize protein bands. Finally, band exposure and analyses were conducted.

### 2.13 Co‐Immunoprecipitation (Co‐IP) Experiments

Co‐IP was performed using a Co‐IP Kit (Thermo Fisher Scientific, 88,804, CA, USA). The total protein was extracted using IP lysis/wash buffer. Immune complexes were prepared according to the manufacturer's instructions. After centrifugation, the supernatant was collected. The supernatant was incubated with anti‐MDM2 (Affinity, AF0208) and anti‐GNL3L (Affinity, DF4113), along with Protein A/G Magnetic Beads (MedChemExpress, USA) overnight at 4°C. Finally, the protein–protein complexes were subjected to a western blot.

### 2.14 Animal Experiment‐Xenograft ESCC Tumor Model

A mouse model of ESCC was created using 18 4‐week‐old BALB/C male nude mice (SPF) from Jinan Pengyue Laboratory Animal Breeding Co Ltd. After sh‐NC and sh‐GNL3L were transfected into TE‐1 cells, the cells (2.5 × 10^6^) were infused subcutaneously into the right axilla of the nude mice [21]. Tumor diameters were measured, and tumor volumes were calculated on a weekly basis. The mice were sacrificed after 5 weeks to weigh the tumors. Hematoxylin and eosin (HE) and terminal deoxynucleotidyl transferase dUTP nick‐end labeling (TUNEL, Procell, P‐CA‐007, Wuhan, China) assays for the transplanted tumors were performed. The study was approved by the Animal Care and Use Committee of Shandong University and the Ethics Committee of Qilu Hospital of Shandong University (Qingdao).

### 2.15 Statistical Analysis

All data analyses were performed using GraphPad Prism 7 (GraphPad Software Inc., San Diego, CA, USA) and SPSS 16.0 (SPSS Inc., Chicago, IL, USA). All experiments were performed in triplicate. The continuous variables with a normal distribution were expressed as means ± standard deviations and compared using Student's *t*‐test (two groups) or one‐way analysis of variance (ANOVA) (three or more groups). Kruskal–Wallis single‐factor ANOVA analysis was used when the continuous variables did not meet the normal distribution or homogeneity of variance. *p* < 0.05 was considered statistically significant.

## 3 Results

### 3.1 *GNL3L* was Highly Expressed in ESCC Specimens and Predicted a Poor Prognosis

**FIGURE 1:**
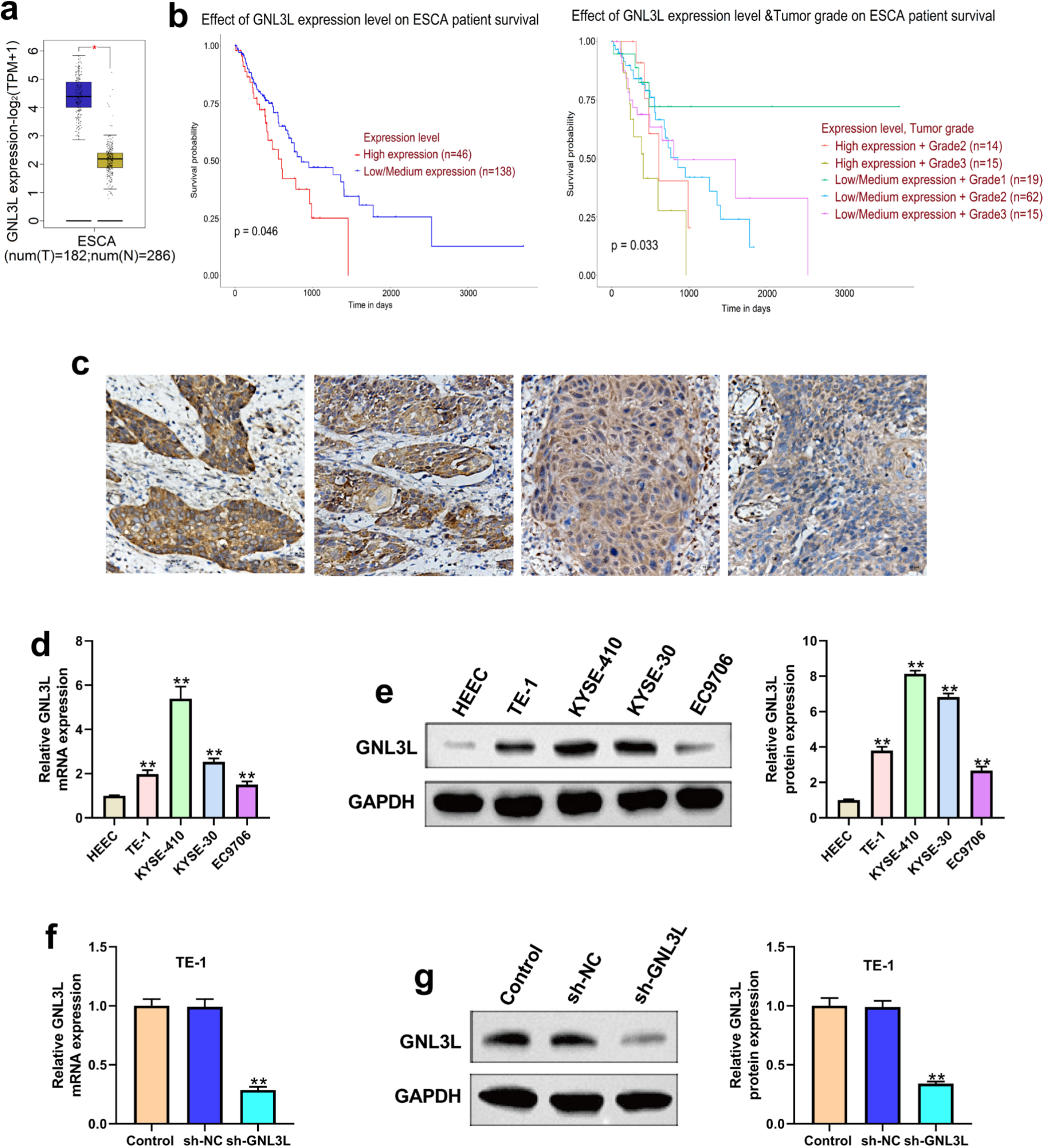
Expression of GNL3L in esophageal cancer (EC) and the cell line model of *GNL3L* knockdown. (a) *GNL3L* expression was analyzed in EC tumor tissues (*n* = 182) and normal esophageal tissues (*n* = 286) using the GEPIA database. (b) Kaplan–Meier analysis of survival rates between patients with EC and low expression of *GNL3L* (*n* = 138) vs. high expression of *GNL3L* (*n* = 46). In addition to the expression of *GNL3L*, the survival rate was also related to tumor grade. The patient cohort was obtained from the gene information of TCGA Esophageal cancer datasets downloaded from the UALCAN website. (c) Immunohistochemistry was performed to detect differential expression of GNL3L protein in an esophageal squamous cell carcinoma (ESCC) specimen. (d) qRT‐PCR was used to detect *GNL3L* expression in ESCC cell lines (TE‐1, KYSE‐410, KYSE‐30, and EC9706) and human normal esophageal epithelial cell line (HEEC). (e) The expression of GNL3L protein in ESCC cell lines and HEEC was assessed using western blotting (WB). (f) Efficiency of sh‐GNL3L for *GNL3L* in TE‐1 cells by qRT‐PCR. (g) The protein expression of GNL3L in TE‐1 cells. (NC: Negative control). (*p < 0.05, **p < 0.01).

According to the TCGA EC datasets, the expression of *GNL3L* in EC tumor tissues was greater than that in normal esophageal tissues (Figure 1a) [22, 23]. Kaplan–Meier analysis showed a better survival probability for EC patients with a lower expression of *GNL3L* (*n* = 138) than those with a higher expression of *GNL3L* (*n* = 46) (Figure 1b). In addition, the expression of *GNL3L* and survival probability were related to tumor grade (Figure 1b) [8, 17]. The patient cohort was obtained from the gene information of the TCGA EC datasets downloaded from the GEPIA and UALCAN websites. The expression of GNL3L in the patients at the authors' hospital was upregulated from strong to negative in ESCC tissues (Figure 1c), with 15 patients showing high expression, 16 showing moderate expression, 13 showing weak expression, and 10 showing no staining. Next, we assessed the relationship between GNL3L expression and clinicopathological characteristics of ESCC patients (Table 1). The results showed that high expression of GNL3L was significantly associated with lymph node metastasis in ESCC patients (*p* = 0.011) and distant metastasis (*p* = 0.008). GNL3L was upregulated in ESCC cell lines compared to HEEC, especially the TE‐1, KYSE‐410, and KYSE‐30 cell lines (Figure 1d,e). The *GNL3L* stable knockdown model was successfully performed using the TE‐1 cell line and measured by qRT‐PCR and western blot, and the efficiency of sh‐GNL3L in TE‐1 cells was approximately 70% (Figure 1f,g).

**TABLE 1:** Association between GNL3L expression and clinicopathological characteristics in esophageal squamous cell carcinoma (ESCC) patients.

Variables	All cases	Low expression of GNL3L		High expression of GNL3L		*p*
Age (years)						0.910
≤ 60	23	10			13	
> 60	31	13			18	
Gender						0.460
Female	4	1			3	
Male	50	22			28	
TNM stage						0.846
I+ II	25	11			14	
III + IV	29	12			17	
Lymph node metastasis						0.011^*^
Yes	34	10			24	
No	20	13			7	
Distant metastasis						0.008^**^
Yes	30	8			22	
No	24	15			9	
Histological differentiation						0.107
High/middle	26	14			12	
Low	28	9			19	

^*^ *p* < 0.05.

^**^ *p* < 0.01.

### 3.2 The Downregulation of *GNL3L* Inhibited the Viability, Migration, and Invasion and Promoted the Apoptosis of ESCC Cells

**FIGURE 2:**
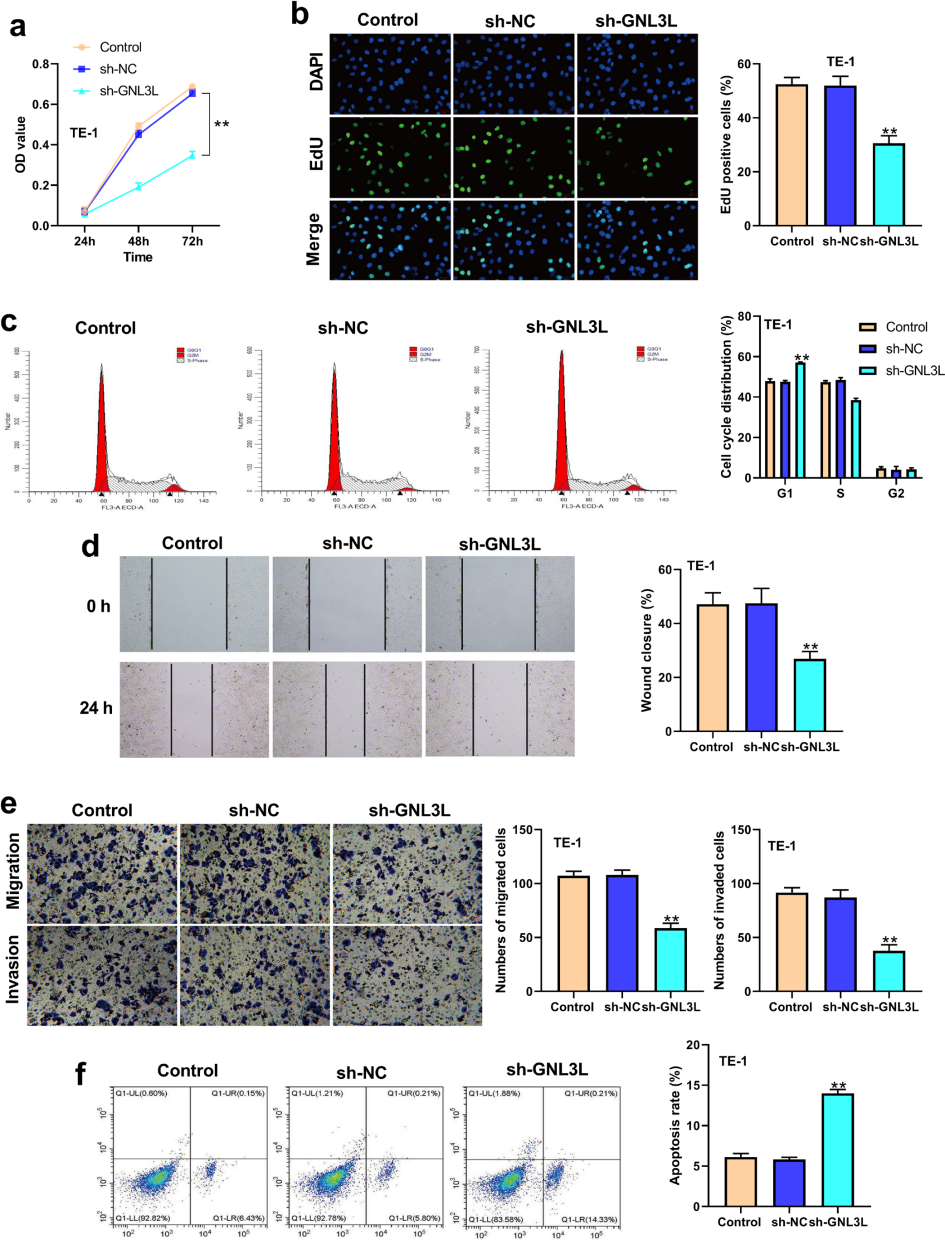
The downregulation of *GNL3L* inhibited the malignant phenotypes in TE‐1 cell lines, including proliferation, invasion, migration, and apoptosis. (a) Cell Counting Kit‐8 (CCK8) assays and (b) EDU assays were performed to analyze cell proliferation in the TE‐1 cell line with sh‐GNL3L, sh‐NC, and Control. (c) Distribution of cell phases of TE‐1 in the different groups. (d) Wound healing assay results in the TE‐1 cell line with sh‐GNL3L, sh‐NC, and Control. (e) Invasion and migration of TE‐1 cells were assessed by Transwell assays. (f) Results of apoptosis using flow cytometry for the TE‐1 cell line in different groups (**p < 0.01).

According to the CCK8 tests, the OD values of sh‐GNL3L, sh‐NC, and Control were 0.18, 0.45, and 0.49 after 48 h and 0.3, 0.6, and 0.62 after 72 h (*p* < 0.01) (Figure 2a). The EdU experiments also showed that *GNL3L* knockdown significantly slowed the proliferation of TE‐1 cells (Figure 2b). The cells were blocked in the G1 phase of TE‐1 cells after the knockdown of *GNL3L* (Figure 2c). According to the wound healing assay results, TE‐1 cells had a smaller healing wound width than sh‐NC and Control group cells when *GNL3L* expression was downregulated (Figure 2d). In addition, the Transwell assay showed that when *GNL3L* expression was downregulated, the migrated and invasive abilities of TE‐1 cells were inhibited (Figure 2e). A significant increase in the percentage of apoptosis in ESCC cells was observed when *GNL3L* expression was downregulated (Figure 2f).

**FIGURE 3:**
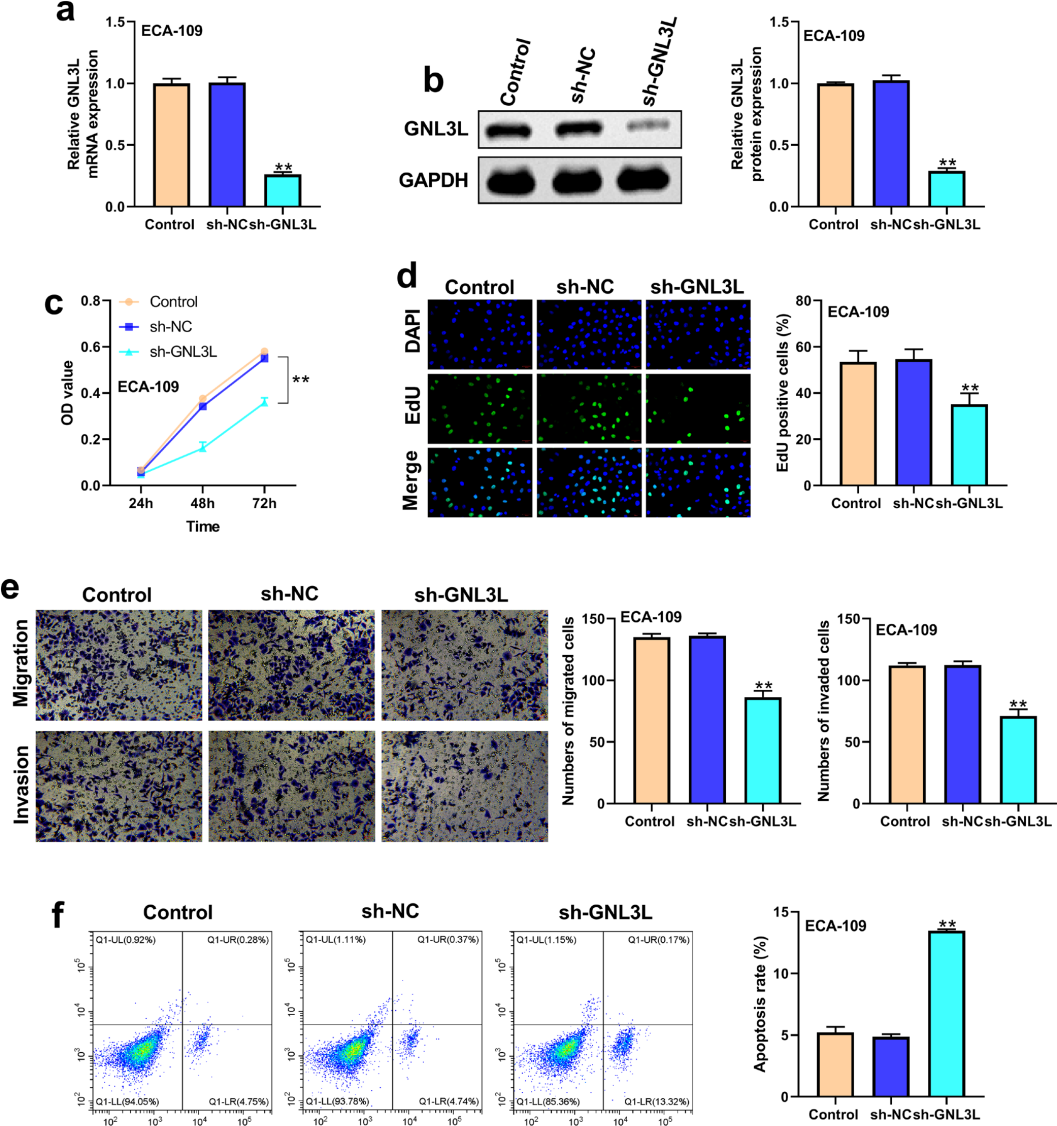
The downregulation of *GNL3L* inhibited the malignant phenotypes in ECA‐109 cell lines, including proliferation, invasion, migration, and apoptosis. (a) Efficiency of sh‐GNL3L for *GNL3L* in ECA‐109 cells by qRT‐PCR. (b) The protein expression of GNL3L in ECA‐109 cells. (c) CCK8 assay and (d) EDU assay were performed to analyze cell proliferation in the ECA‐109 cell line with sh‐GNL3L, sh‐NC, and Control. (e) Migration and invasion of ECA‐109 cells were assessed by Transwell assays. (f) Results of apoptosis using flow cytometry for the ECA‐109 cell line in different groups (**p < 0.01).

The transfection efficiency of sh‐GNL3L in ECA‐109 cells was verified by using qRT‐PCR and western blot (Figure 3a,b). The results of the CCK8 assay showed that the OD values of sh‐GNL3L, sh‐NC, and Control were 0.16, 0.34, and 0.37 after 48 h and 0.36, 0.55, and 0.58 after 72 h (*p* < 0.01) (Figure 3c). The results of the EdU assay also showed that *GNL3L* knockdown significantly inhibited the proliferation of ECA‐109 cells (Figure 3d). Moreover, the migrated and invasive abilities of ECA‐109 cells were inhibited after the knockdown of *GNL3L* (Figure 3e). Besides, the apoptosis of ECA‐109 cells was increased after the knockdown of *GNL3L* (Figure 3f).

### 3.3 GNL3L Positively Regulated MDM2 Expression and Interacted With MDM2

**FIGURE 4:**
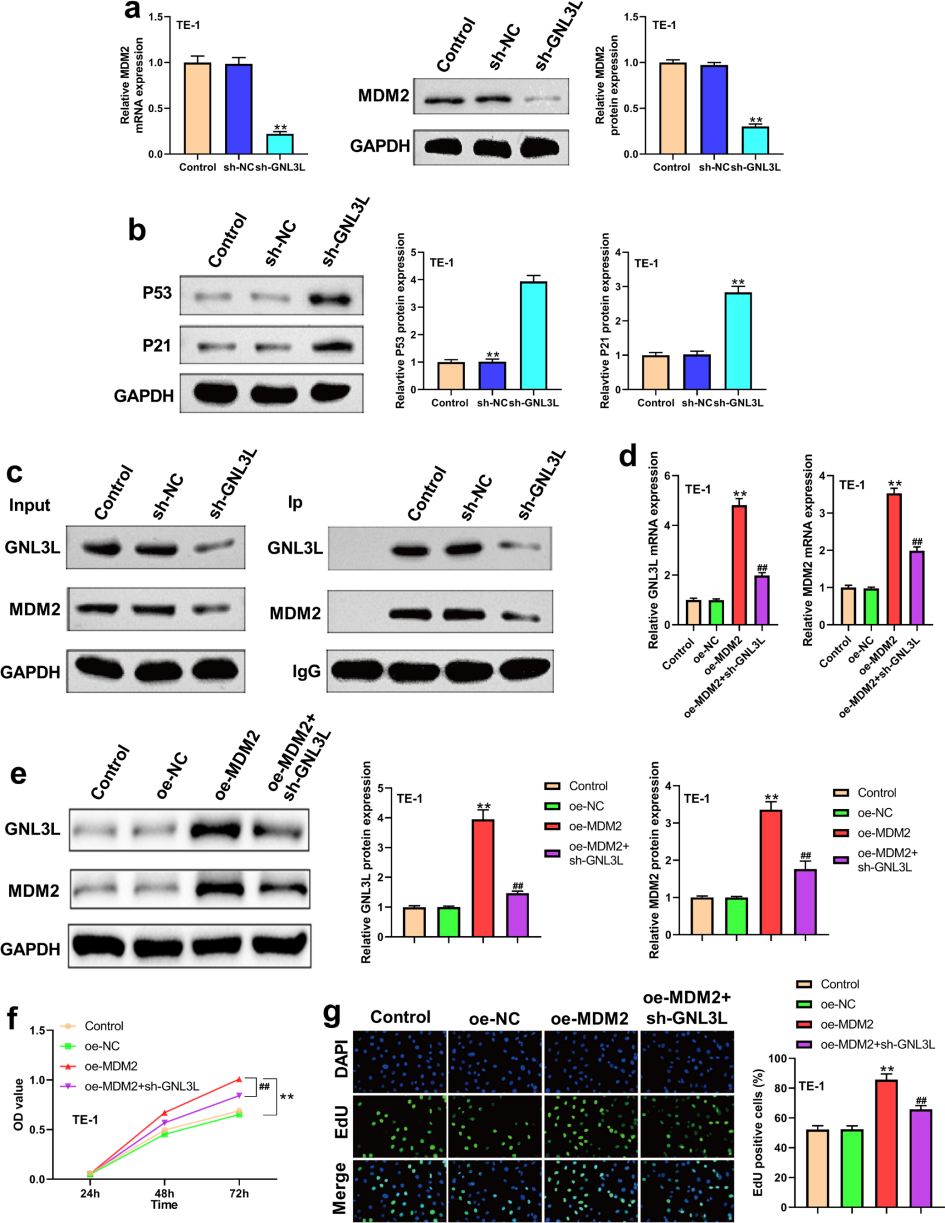
GNL3L positively regulated MDM2 expression and interacted with MDM2. (a) After *GNL3L* knockdown, qRT‐PCR, and western blot (WB) were performed to determine MDM2 expression in TE‐1 cells. (b) Differences in the expression of p53 and p21 in TE‐1 cells after the knockdown of GNL3L. (c) Co‐IP of GNL3L and MDM2 from ESCC cell lines. The input represents the total protein extract used in the IP. The GNL3L protein expression was normalized to that of GAPDH in ESCC cells. (IP, immunoprecipitation; IgG, negative control). (d) qRT‐PCR analysis of *MDM2* and *GNL3L* expression in TE‐1 cells in different groups. (e) Protein expression of MDM2 and GNL3L of TE‐1 in different groups was determined by WB. (f) Cell proliferation was analyzed in different groups using the CCK8 assay for TE‐1 cell lines. (g) Proliferation was analyzed using an EdU assay. (**p < 0.01, ^##^ *p* < 0.01).

After *GNL3L* knockdown, MDM2 mRNA and protein levels were downregulated (Figure 4a), whereas p53 and p21 levels were upregulated in TE‐1 cells (Figure 4b). Co‐IP experiments showed that GNL3L interacted with MDM2 (Figure 4c). Additionally, MDM2 overexpression increased the GNL3L mRNA and protein expression (Figure 4d,e).

### 3.4 GNL3L Interacts With MDM2 to Determine the Malignant Phenotypes of ESCC Cells

**FIGURE 5:**
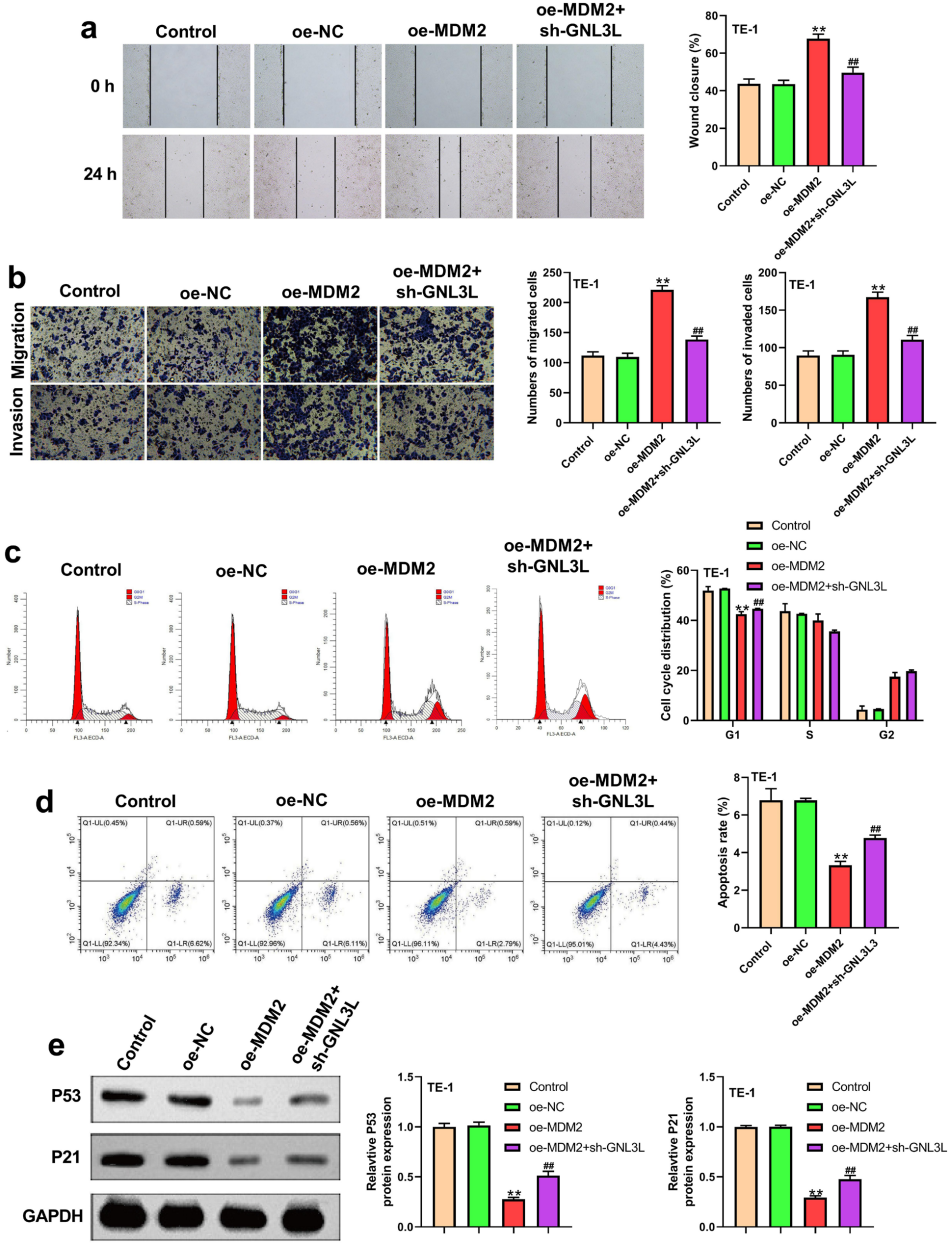
*GNL3L* knockdown reversed the increased malignant behaviors of ESCC cells induced by *MDM2* overexpression. (a) Wound healing assay results in TE‐1 cells from different groups. (b) Invasion and migration of TE‐1 cells were assessed using Transwell assays. (c) Distribution of the cell phases of TE‐1 in the different groups. (d) In the different groups, apoptosis was measured in TE‐1 cells using flow cytometry. (e) Expression of P53 and P21 in TE‐1 cells in different groups. (**p < 0.01, ^##^ *p* < 0.01).

The overexpression of *MDM2* resulted in higher cell proliferation, invasion, and migration, whereas co‐transfection with oe‐MDM2 and sh‐GNL3L reversed these phenotypes (Figure 4, g and Figure 5, b). Co‐transfection of oe‐MDM2 and sh‐GNL3L in ESCC cells blocked the distribution of cells in the G1 phase (Figure 5c). Similarly, *MDM2* overexpression inhibited apoptosis, and co‐transfection with oe‐MDM2 and sh‐GNL3L enhanced the percentage of apoptotic cells (Figure 5d). p53 and p21 were downregulated in ESCC when *MDM2* was overexpressed, whereas they were upregulated when the cells were co‐transfected with oe‐MDM2 and sh‐GNL3L (Figure 5e). Hence, the overexpression of *MDM2* increased the malignant phenotype of ESCC cells, whereas the concomitant downregulation of *GNL3L* reversed the effects of *MDM2* overexpression.

**FIGURE 6:**
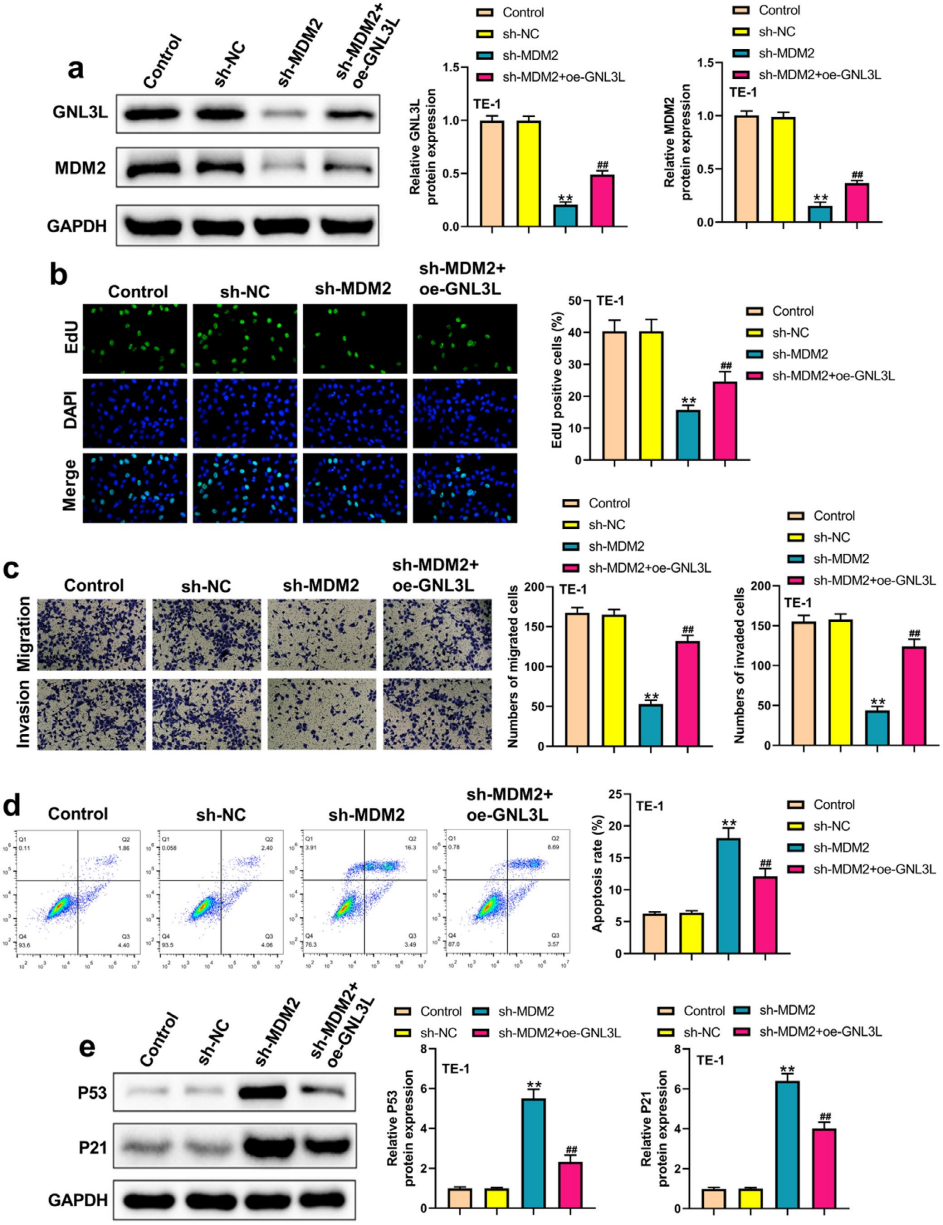
*GNL3L* overexpression reversed the decreased malignant behavior of ESCC cells induced by *MDM2* knockdown. (a) Protein expression of MDM2 and GNL3L in TE‐1 cells in different groups was determined by western blot (WB). (b) Cell proliferation was analyzed in different groups using an EdU assay. (c) Migration and invasion of TE‐1 cells were assessed using Transwell assays. (d) In the different groups, apoptosis was measured in TE‐1 cells using flow cytometry. (e) Expression of P53 and P21 in TE‐1 cells in different groups. (**p < 0.01, ^##^ *p* < 0.01).

Moreover, *MDM2* knockdown decreased the GNL3L protein expression (Figure 6a). *MDM2* knockdown significantly decreased the proliferation, invasion, and migration of TE‐1 cells, whereas GNL3L overexpression reversed these phenotypes (Figure 6, c). Meanwhile, *MDM2* knockdown increased the apoptosis of TE‐1 cells, whereas GNL3L overexpression significantly reversed the increased apoptosis (Figure 6d). As shown in Figure 6e, GNL3L overexpression significantly reversed the upregulated expression of p53 and p21 in TE‐1 cells induced by MDM2 knockdown. All these data indicated the role of GNL3L in the effects of MDM2.

### 3.5 The Downregulation of *GNL3L* Inhibited the Growth of ESCC in a Xenograft Model

**FIGURE 7:**
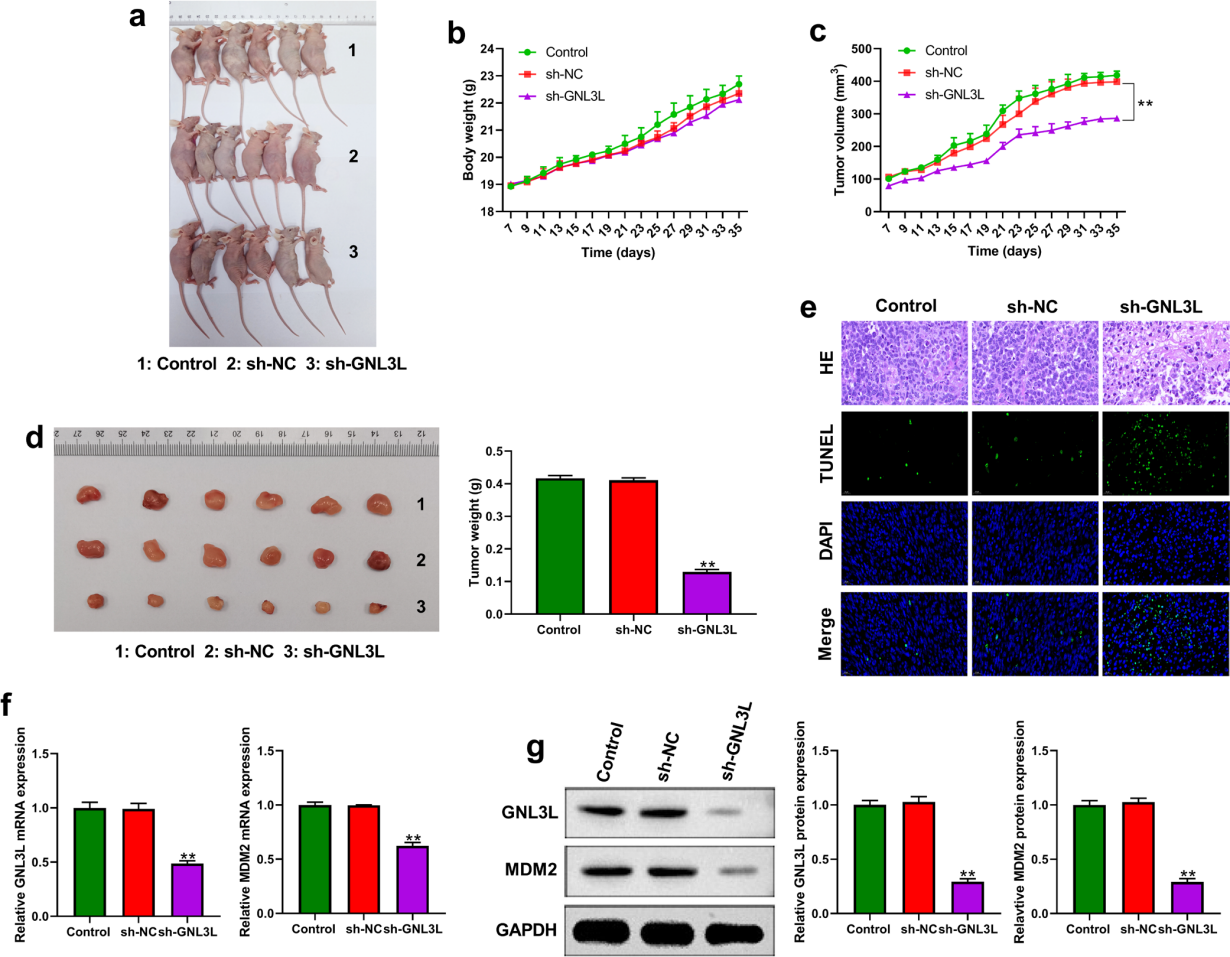
Tumor formation experiment in nude mice after knockdown of *GNL3L*. (a) Three groups of tumor cells were implanted in the right axilla of nude mice. (b) Changes in body weight of three groups of nude mice. (c) Tumor volume of the three groups of nude mice. (d) The size of transplanted tumors in three groups of nude mice. (e) HE and TUNEL assays for the transplanted tumor of the three groups. (f) The mRNA expression of *GNL3L* and *MDM2* for the transplanted tumor of three groups with qRT‐PCR. (g) The protein expression of GNL3L and MDM2 for the transplanted tumor of three groups with western blotting. (**p < 0.01).

Then, the in vitro results were confirmed in vivo. Nude mice were inoculated with TE‐1 cells expressing or not *GNL3L*. After 5 weeks, the mice were sacrificed, and the sh‐GNL3L group had the smallest tumor volumes (Figure 7a). The tumors were weighed, and the sh‐GNL3L group had the lightest tumor weights and volumes (Figure 7, d). HE and TUNEL assays for the transplanted tumors of three groups were performed, showing that the percentage of apoptotic cells of the sh‐GNL3L group was the highest (Figure 7e). In the sh‐GNL3L group, the mRNA and protein expression of GNL3L and MDM2 were lower than in the other groups (Figure 7, g).

## 4 Discussion

This study examined the mechanisms underlying the role of *GNL3L* in ESCC progression. The results suggest that *GNL3L* expression is associated with the ESCC malignant phenotype. *GNL3L* knockdown downregulates MDM2 and upregulates p53 and p21. *MDM2* overexpression increases the malignant phenotype, which is reversed by the simultaneous silencing of *GNL3L*.

ESCC is an invasive and heterogeneous malignancy with a complicated etiopathogenesis [24]. The inactivation of tumor suppressors and the activation of oncogenes by various signaling pathways play crucial roles in the development of ESCC [25, 26]. The identification of novel biomarkers for ESCC treatment and diagnosis is essential.

*GNL3L* is predominantly found in the nucleolus and the nucleoplasm [27, 28]. The nuclear localization of *GNL3L* promotes the binding of cyclin D1‐CDK4 and might lead to increased phosphorylation of the serine 780 site of the *Rb* gene [27, 28]. *GNL3L* promotes S phase progress and tumor cell proliferation by regulating the Rb‐E2F1 pathway [29, 30]. Moreover, *GNL3L* overexpression increases tumor cell proliferation [31]. Knockdown of *GNL3L* could cause G2/M phase arrest and regulate specific *p53* targeted proteins in the *p53* wild‐type human colon cancer cell line HCT116 [11]. Accordingly, the inhibition of *GNL3L* in the present study decreased ESCC cell proliferation in vitro and in vivo, supporting the role of *GNL3L* in the pathogenesis of ESCC. The cell cycle was arrested in G1, leading to increased apoptosis after silencing *GNL3L*. In addition, the bioinformatics results and the results from the ESCC specimen support the higher GNL3L expression in ESCC tissues than in adjacent healthy tissues. Those results are supported by Dai et al. [8].

Nevertheless, the molecular mechanisms linked to *GNL3L* and the aggressiveness of ESCC remain mostly unknown. It has been reported that the knockdown of leucine zipper downregulated in Cancer‐1 (*LDOC1*) induced cell proliferation by upregulating *GNL3L*. The interaction between *GNL3L* and *LDOC1* regulates cell proliferation via the NF‐κB pathway [32]. Other researchers have found that *miR‐4454* is a key precursor of the post‐transcriptional inhibition of the *GNL3L* gene in colon cancer progression, and silencing *GNL3L* significantly reduced the survival of colon cancer cells [7]. Meng et al. [11] showed that GNL3L can bind MDM2 and stabilize the MDM2 protein to prevent its ubiquitination and degradation. Accordingly, in the present study, the Co‐IP assay showed that GNL3L can bind MDM2. In addition, the overexpression of *MDM2* increased the aggressive phenotype of ESCC cells. Still, the simultaneous silencing of *GNL3L* reversed the effects of overexpressing *MDM2*. Moreover, GNL3L overexpression reversed the decreased malignant behavior of TE‐1 cells induced by MDM2 knockdown. These data suggest that the absence of GNL3L could not prevent MDM2 ubiquitination and that MDM2 was degraded. The silencing of *GNL3L* increased the expression of p53 and p21, which are important tumor suppressor genes [15].

*GNL3L* has been associated with tumor mutation burden and microsatellite instability in various cancers [33]. In addition, clustered heat maps demonstrated a positive correlation between *GNL3L* and CD4^+^ T cell memory, particularly in EC [33]. Therefore, high levels of *GNL3L* may affect immune checkpoint inhibitor treatment, which could have clinical implications for the management of EC. The *p53* gene is a negative regulator of the cell growth cycle and a tumor suppressor [15]. In addition to regulating the cell cycle, DNA repair, differentiation, and apoptosis, it plays an important role in many other biochemical processes [34]. Previous results have indicated that the *p53* status of cancer cells also profoundly impacts the immune response [35]. The E3 ubiquitin ligase MDM2 is a key negative regulator of p53 and plays a crucial role in tumor development and is highly expressed in tumors [36]. *MDM2* is amplified in EC [37, 38], and *MDM2* polymorphisms are associated with EC risk [39]. MDM2 binds to p53 to block its tumor‐inhibitory transactivation domain but also acts as an E3 ligase, which could ubiquitinate p53 and increase its degradation by the proteasome [40]. Nevertheless, additional studies are necessary to examine the interactions of GNL3L, MDM2, p53, and p21 in the pathogenesis of ESCC. Those aspects will have to be examined in future studies.

The present study had limitations. It was performed on a small number of patients. The lack of inhibition and overexpression of the various proteins involved limited the molecular experiments. Future studies should examine each protein involved in the GNL3L‐MDM2‐p53 axis to determine their exact roles in ESCC.

## 5 Conclusions

This study showed that a higher *GNL3L* expression is associated with a malignant phenotype in ESCC. Silencing *GNL3L* decreases the aggressiveness of ESCC. *MDM2* overexpression increases the malignant phenotype of ESCC. *GNL3L* knockdown downregulates MDM2 and upregulates p53 and p21. Future studies should examine how *GNL3L* could be targeted to improve the management of ESCC.

## Author Contributions

**Aijie Yang:** methodology, software, data curation, investigation, formal analysis, visualization, writing – original draft, writing – review and editing. **Haiyun Song:** methodology, software, data curation, investigation, writing – original draft, writing – review and editing. **Yufeng Cheng:** conceptualization, investigation, validation, supervision, funding acquisition, visualization, project administration, resources, writing – review and editing.

## Disclosure

Institutional Review Board Statement: The research complied with all relevant national regulations, institutional policies, and Helsinki Declaration principles; it was approved by the Ethics Committee of Qilu Hospital at Shandong University (Qingdao).

## Conflicts of Interest

The authors declare no conflicts of interest.

## Data Availability

All data generated or analyzed during this study are available from the corresponding author upon reasonable request.
